# Preparation of 1, 3, 6, 8-Pyrenesulfonic Acid Tetrasodium Salt Dye-Doped Silica Nanoparticles and Their Application in Water-Based Anti-Counterfeit Ink

**DOI:** 10.3390/ma13184074

**Published:** 2020-09-14

**Authors:** Liyong Jiao, Mengnan Zhang, Houbin Li

**Affiliations:** 1 School of Printing and Packaging, Wuhan University, Wuhan 430072, China; 2 School of Light Industry and Chemical Engineering, Dalian Polytechnic University, Dalian 116034, China; dicp1808@163.com.cn

**Keywords:** 1, 3, 6, 8-pyrenesulfonic acid tetrasodium salt (PTSA), silica nanoparticles (SiNPs), cationic polyelectrolyte, luminescent properties, water-based anti-counterfeit ink

## Abstract

In order to improve the luminescent stability of water-based anti-counterfeit ink, a new fluorescent material is prepared by doping dye into silica nanoparticles. Water soluble anionic dye 1, 3, 6, 8-pyrenesulfonic acid sodium salt (PTSA) is selected as the dopant. In this work, PTSA is successfully trapped into silica nanoparticles (SiNPs) by the reverse microemulsion method using cationic polyelectrolyte poly (dimethyl diallyl ammonium chloride; PDADMAC) as a bridge. The UV absorption spectra, fluorescence emission spectra and fluorescent decay curves are used to describe the luminescent properties of the PTSA-doped silica nanoparticles (PTSA-SiNPs). In addition, the as-prepared PTSA-SiNPs and polyurethane waterborne emulsion are used to prepare water-based anti-counterfeit ink, and fluorescent patterns are successfully printed through screen-printing. The samples printed by the ink exhibit desirable fluorescence properties, heat stability, robust photostability, and a fluorescent anti-counterfeit effect, which makes the PTSA-SiNPs promising luminescent materials for anti-counterfeit applications.

## 1. Introduction

In modern anti-counterfeit printing technology, fluorescent anti-counterfeit ink is widely used in the anti-counterfeit of commercial bills, high-grade commodity packaging and labels because of its convenience in verification and prevention of forgery [1,2]. Graphic messages printed by this ink cannot be seen under visible light, but become visible under excitation using appropriate wavelength light. With the compulsory implementation of green printing standards all over the world, the research of water-based fluorescent anti-counterfeit ink is attracting significant attention [3].

The components of water-based anti-counterfeit ink include binders, pigments, solvents and additives. The anti-counterfeit function of the ink mainly depends on fluorescent materials as pigments. Therefore, the fluorescent materials in the ink not only have good fluorescence performance, but also have good fluorescence stability, which is not affected by other components of ink, the system environment, processing conditions and other factors.

In the past several years, various fluorescent materials have been explored in the preparation of water-based anti-counterfeit ink, such as organic fluorescent dyes [4,5], rare-earth luminescent materials [6,7,8], carbon dots [9,10,11,12], organic polymers [13,14] etc. Compared with other fluorescent materials, organic fluorescent dyes have the advantages of easy access to raw materials, more variety, bright colors and a low cost. 

The typical water-based anti-counterfeit ink usually contains a small amount of water-soluble fluorescent dyes due to the concentration quenching phenomenon, so it is difficult to improve the fluorescence intensity. Moreover, the ink contains a good solubility of fluorescent dyes, which can prevent aggregation and association, and obtain strong fluorescence intensity. However, due to the weak environmental adaptability of dyes, the fluorescent stability of printing image is deteriorated. Some effective research has been achieved by using dye-doped silica nanoparticle (SiNP) material in water-soluble anti-counterfeit ink [15,16]. Thousands of dye molecules mingle with silica matrix, which can protect the dye from the surrounding environment, improve luminescent stability and chemical stability [17,18], provide signal enhancement [19,20], and solve the toxicity of organic fluorescent dye.

To date, dye-doped SiNPs are mainly prepared by three methods: the covalent coupling method, the electrostatic adsorption method based on the Stöber system [21,22,23,24], and the reverse microemulsion method [25,26,27]. The reverse microemulsion system is a water/oil type microemulsion, which is composed of an oil-continuous phase, a water-dispersed phase, a surfactant and a cosurfactant. Thus, it is very suitable for preparing water-soluble dye-doped SiNPs. The water is surrounded by the interface composed of the surfactant and cosurfactant, and it forms a 10–100 nm nanoscale space with uniform size. Microemulsion is a thermodynamic stable system that provides an ideal microenvironment for the preparation of uniformly sized nanoparticles. These nanoparticles can be easily manipulated by varying microemulsion parameters with regard to the particle size and shape. Various nanoparticles suitable for different applications have been prepared by this method, showing its flexibility for the fabrication of different types and sizes of silica nanoparticles. 

1, 3, 6, 8-pyrenesulfonic acid tetrasodium salt (PTSA) is a typical water-soluble anionic fluorescent dye. Because of its excellent fluorescence properties [28,29], such as clear spectrum, fine structure, extremely response to micro-environment etc., PTSA has drawn considerable interest and is widely used in the imaging, labelling [30], biomedical [31], and industrial fields [32]. However, it also has disadvantages similar to other fluorescent dyes. It can also be easily degraded and oxidized because of its electron transition under UV light. These problems affect its application quality and field. At present, there are few reports regarding anionic fluorescent dye-doped SiNPs and their application.

In this study, PTSA-doped silica nanoparticles (PTSA-SiNPs) are prepared by the reverse microemulsion method using cationic polyelectrolyte poly (dimethyl diallyl ammonium chloride) (PDADMAC) as a bridge. The luminescent properties of PTSA-SiNPs are investigated by UV absorption spectra, fluorescence emission spectra and fluorescent decay curves. The as-prepared PTSA-SiNPs are applied in the formulation of water-based fluorescent anti-counterfeit ink. The samples printed by the ink have a good anti-counterfeit effect and fluorescence stability, and, notably, they show excellent light resistance and heat resistance, providing a potential and high-quality blue fluorescent material for application in water-based ink. 

## 2. Materials and Methods 

### 2.1. Materials

PTSA was prepared by our laboratory [5]. Triton X-100 and n-hexanol were from Tianjin Guangfu Fine Chemical Research Institute (Tianjin, China). Cyclohexane, tetraethyl orthosilane (TEOS), ammonia (25%), ethanol and acetone were from Tianjin Kemio Chemical Reagent Co., Ltd. (Tianjin, China). The above chemical reagents were of analytical grade and were not purified before use. PDADMAC was from Wuxi Tianxin Chemical Co., Ltd. (Wuxi, China). Polyurethane waterborne emulsion was from ATOZ (China) Fine Chemical Co., Ltd. (Tianjin, China). The non-background papers free of fluorescent agent were purchased from Ningbo Zhonghua Paper Co., Ltd. (Ningbo, China). High-purity water (Pall Purelab Plus, New York, NY, USA) with a resistivity of 1.8 × 10^5^ Ω·m was used for the preparation of all aqueous solutions.

### 2.2. Preparation of PTSA-SiNPs Using the Reverse Microemulsion Method 

First, a proper amount of 2 mg/mL of PDADMAC aqueous solution was prepared at room temperature; according to the ratio of n_anion_:n_cation_ = 0.53:1, a certain amount of PTSA was weighed and dispersed into PDADMAC aqueous solution, stirring the solution until PTSA was completely dissolved into the solution. Finally, the PTSA/PDADMAC complex solution was obtained.

PTSA-SiNPs were achieved as reported in the literature as follows [33]: Triton X-100 (1.77 mL), hexanethol (1.80 mL) and cyclohexane (7.5 mL) were mixed into a 50 mL three port round bottom flask. The mixture was stirred uniformly at room temperature for 30 min; then, the PTSA/PDADMAC complex solution (100 µL) was dripped into the mixture. After the reaction mixture was further stirred for 1 h, TEOS (200 µL) was slowly added to the microemulsion system and stirred for 30 min; finally, ammonia (120 µL) was added to catalyze the reaction, which needed to be stirred for 24 h with the temperature maintained at 25 °C until the end. The product was obtained by adding acetone under centrifugation. Then, it was purified with anhydrous ethanol and ultrapure water 3 to 4 times to remove unreacted materials. Finally, the purified product was vacuum dried. The PTSA solution with the same concentration was added into the reaction system to prepare PTSA-SiNPs as a comparative experiment.

### 2.3. Screen Printing Process of the PTSA-SiNPs Anti-Counterfeit Ink 

The anti-counterfeit ink is composed of pigment, binder, solvent and additives. The as-prepared PTSA-SiNPs are the pigment, and polyurethane dispersion emulsion is the binder. The solvent is the mixed solution of water and ethanol in order to adjust viscosity and speed up the drying speed of water-based ink. Additives mainly include a defoamer and levelling agent. The formula of the PTSA-doped SiNP (PTSA-SiNP) ink and PTSA ink can be seen in Table 1. At first, the PTSA-SiNPs were mixed with the polyurethane dispersion emulsion. The process was carried out for 1 h on a magnetic stirrer. Later on, solvent and additives were added successively and stirred continuously until a homogeneous viscous ink was obtained. The screen-printing plate was based on 200 meshes of polyester fiber. The non-background paper was chosen as a printing substrate. 

### 2.4. Dye Leakage Test 

Anti-leakage ability is an important index to evaluate the quality of dye-doped SiNPs. The purified PTSA-SiNPs were dissolved in ultrapure water, and evenly dispersed under an ultrasonic condition. Sample solution (3 mL) was taken within 0 h, 6 h, 12 h, 24 h and 240 h. The supernatant was discarded after high-speed centrifugation, and then the PTSA-SiNPs were dispersed again in ultrapure water to obtain uniform suspension. The fluorescent intensity was measured by fluorescence spectrophotometer under the excitation wavelength of 376 nm. The leakage degree of PTSA was determined by the change in fluorescent intensity.

### 2.5. Photostability Test 

The anti-counterfeit ink was printed on the paper and cut into small pieces. The pieces samples were irradiated twice under ultraviolet light (354 nm) for 30 min each time. Then, the maximum fluorescent intensity was measured by a fluorescence spectrometer. Each sample was tested three times, and its average value was taken as the result. 

### 2.6. Heat Stability Test 

The manufacture method of the test samples is the same as the photostability test. The pieces samples were put into the electric blast drying oven which was heated for 30 min. The heating temperature was 40 °C, 60 °C, 80 °C and 100 °C, respectively. The data processing method is the same as that of the photostability test.

### 2.7. Characterization

Transmission electron microscopy (TEM) images were observed under a JEOL-2010 electron microscope (JEOL, Tokyo, Japan) operating at 200 kV. Scanning electron microscopy (SEM) and energy dispersive spectroscopy (EDS) was performed on a JSM-7800F field-emission scanning electron microscope (JEOL, Tokyo, Japan) with an X-Max50 energy spectrometer (Oxford Instruments, Oxford, UK). Nanoparticle size and zeta potential were determined using a Malvern Zetasizer 3000HSA instrument (Malvern Instruments, Malvern, UK). Fourier transform infrared spectra (FT-IR) were obtained on a spectrum two infrared spectrometer (Perkinelmer, Walsham, MA, USA) using pressed KBr discs. UV absorption spectra were carried out using a LAMBDA 35 spectrophotometer (Perkinelmer, Walsham, MA, USA). The fluorescent spectra were recorded on a LS-55 fluorescence spectrometer (Perkinelmer, Walsham, MA, USA). Luminescence decay curves were obtained using an FLS-980 fluorescence spectrometer (Edinburgh Instruments, Scotland, UK). The excitation light source for obtaining the fluorescence photos was a ZF1-2 UV analyzer (Shanghai Precision Instrument, Shanghai, China) with UV light (254 nm and 365 nm) and simulated sunlight.

## 3. Results and Discussion

### 3.1. Preparation and Characterization 

#### 3.1.1. Preparation Mechanism of PTSA-SiNPs 

**Figure 1:**
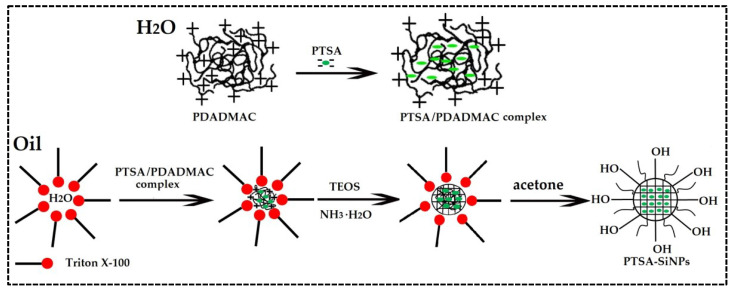
The preparation mechanism diagram of PTSA-SiNPs.

The preparation mechanism of PTSA-SiNPs is displayed in Figure 1. PTSA is dissociated into anions in aqueous solution. The surface potential of SiNPs is negative. Based on the principle that the same electrical properties repel each other, it is not easy to dope PTSA directly into SiNPs. PDADMAC is a kind of water-soluble cationic polyelectrolyte, which has the characteristics of high positive-charge density, good water solubility, high efficiency, non-toxicity and wide pH application. Therefore, PDADMAC is chosen as the intermediate between PTSA and SiNPs to prepare PTSA-SiNPs. Firstly, the PTSA/PDADMAC complex solution is prepared by dissolving PTSA in PDADMAC aqueous solution. The molar ratio of n_anion_:n_cation_ in the PTSA/PDADMAC complex system can be controlled in the range of 0.2–0.7:1. In this paper, the ratio of n_anion_:n_cation_ is 0.53:1. In the microemulsion system, the aqueous phase is divided into many “droplets” in the organic phase by surfactant Triton X-100. When the PTSA/PDADMAC complex is dissolved in these droplets, a microemulsion droplet containing a hydrophilic core material is formed. Then, silicon source material TEOS is added into the system, hydrolyzed and condensed under the action of basic catalyst ammonia water, and wrapped on the surface of the hydrophilic core. Finally, acetone is added to demulsify in order to obtain the product.

The product is a white solid powder, while PTSA is a light yellow solid powder. The formation of the PTSA-SiNPs is preliminarily determined by comparing the appearance of samples before and after the reaction. The zeta potential of the PTSA/PDADMAC complex solution is about 3.6 mv before the microemulsion reaction. During the reaction, the zeta potential of the PTSA/PDADMAC complex decreased to −17 mv after half an hour of TEOS addition. These data show that negative silicic acid can be adsorbed on the surface of the complex rapidly, and the potential of the complex changes rapidly. With the further growth of SiO_2_ shell, the potential of PTSA-SiNPs remains at −31 mv, which is the reason why PTSA-SiNPs can exist and grow stably in the reaction system. 

The structure of the PTSA-SiNPs prepared with PDADMAC as the intermediate is analyzed by FT-IR spectra (Figure S1b). The absorption peaks at 1098 cm^−1^ and 801 cm^−1^ are assigned to Si-O bond stretching vibrations. Compared with the absorption peak of the typical Si-O bond, there is a red shift, which indicates that the interaction between SiO_2_ and the PTSA/PDADMAC complex molecules affects the vibration of the Si-O bond. The absorption peak at 554 cm^−1^ is assigned to the in-and-out of plane deformation vibration of the benzene ring. The weak peak at 2926 cm^−1^ is assigned to =C-H bond stretching vibration of the benzene ring originating from PTSA. EDX spectra are used to qualitatively determine the elemental compositions of the PTSA-SiNPs (Figure S2a). The elements and weight distribution of C, O, Si and S can be found, respectively. Si and O mostly come from silica nanoparticles. C and S mainly originate from PTSA. This suggested that the resultant product contains these elements. FT-IR (Figure S1c) and EDX (Figure S2b) spectra of the product prepared without using PDADMAC do not show obvious doping characteristics. These results illustrate that PTSA has achieved doping into silica nanoparticles by using PDADMAC as the intermediate. 

#### 3.1.2. Morphology 

**Figure 2:**
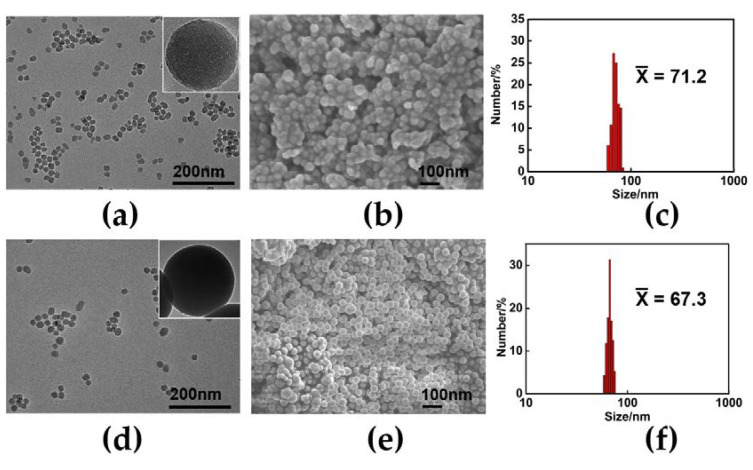
(**a**) TEM and (**b**) SEM images of the PTSA-SiNPs prepared with cationic polyelectrolyte poly (dimethyl diallyl ammonium chloride) (PDADMAC) as the intermediate; (**c**,**f**) size distribution of the PTSA-SiNPs prepared with and without PDADMAC as the intermediate, respectively; (**d**) TEM and (**e**) SEM images of the PTSA-SiNP prepared without PDADMAC as the intermediate.

The morphology of the PTSA-SiNPs prepared with and without PDADMAC as an intermediate can be characterized by TEM and SEM, as shown in Figure 2. Figure 2a,d show their TEM image. Particle morphologies of two products are regular, spherical, smooth on the surface, uniform in size and good in dispersion. Figure 2b,e demonstrate their SEM images. The results of SEM show that the morphology of the two products is similar to that of TEM, respectively. As shown in Figure 2c,f, the particle size of the two products measured by dynamic light scattering (DLS) radius in ethanol is 67.3 nm and 71.2 nm, respectively. The size obtained by DLS is usually large because of surrounding solvent molecules and the swelling of surface molecules [34]. Generally, the smaller the particle size, the greater the stability of the dispersion. The results show that the morphology and size distribution of the product particles prepared by the reverse microemulsion method are in line with our expectations, and are suitable to be applied to polydisperse systems, such as in printing ink.

#### 3.1.3. UV Absorption and Optical Property

**Figure 3:**
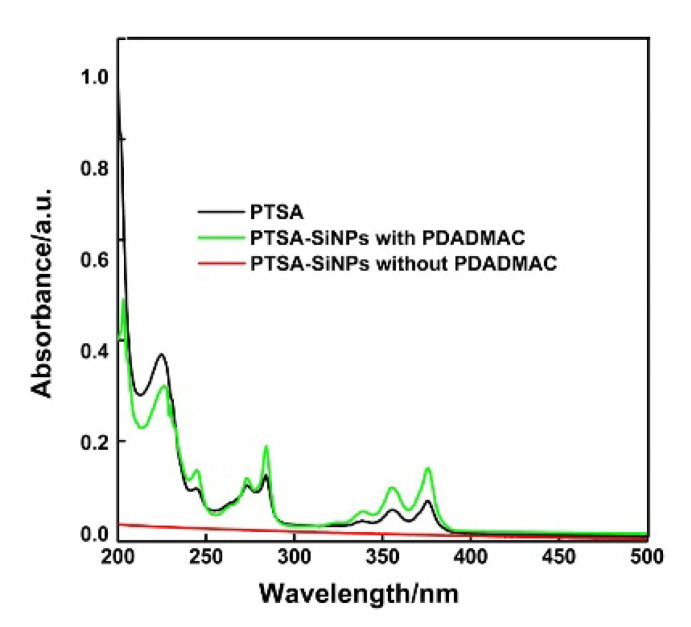
UV absorption spectra of the original PTSA and PTSA-SiNPs prepared with and without PDADMAC as the intermediate in H_2_O (concentration 10^−6^ M). The concentration refers to the final concentration of PTSA in the solution. The original PTSA solution was prepared by direct calculation and weighing. PTSA-SiNP suspension solution is prepared based on the absorption spectra of the PTSA at Lambert–Beer law.

UV absorption spectra of the original PTSA and the PTSA-SiNPs prepared with and without PDADMAC as the intermediate in water are shown in Figure 3. The absorption behavior of the dispersion of PTSA-SiNPs prepared with PDADMAC is similar to that of the original PTSA solutions. It has two prominent absorption bands both 285 nm and 378 nm. The absorption peak at 285 nm is due to the π–π* transition of C=C, and the absorption at 378 nm corresponds to the π–π* transition of the C=O bond. These characteristic peaks represent a typical absorption of an aromatic π-system. It is also observed that the dispersion of PTSA-SiNPs prepared without PDADMAC solution has no obvious UV absorption. 

**Figure 4:**
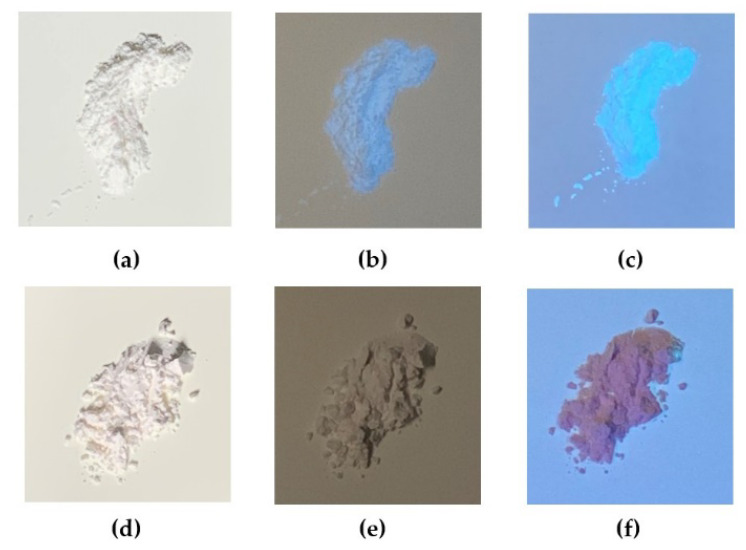
Photos of the PTSA-SiNPs prepared with PDADMAC as the intermediate (**a**) under sunlight, (**b**) 254 nm UV light and (**c**) 365 nm UV light; photos of the PTSA-SiNPs prepared without PDADMAC (**d**) under sunlight, (**e**) 254 nm UV light and (**f**) 365 nm UV light.

The photos of the PTSA-SiNPs prepared with and without PDADMAC as the intermediate under different light conditions are shown in Figure 4. Figure 4a,d show that the two products are white powder materials without fluorescence under sunlight. Under the UV light irradiation of different wavelengths (254 nm and 365 nm), the product prepared with PDADMAC in Figure 4b,c exhibits strong blue fluorescence, while the product prepared without PDADMAC does not show fluorescence phenomenon in Figure 4e,f. The optical property of the solid-state products and the UV absorption of the products in water fully prove that PTSA-SiNPs are successfully prepared by the reverse microemulsion method with PDADMAC as a bridge, and it was difficult to achieve the aim without PDADMAC.

#### 3.1.4. Fluorescence Emission Spectra

**Figure 5:**
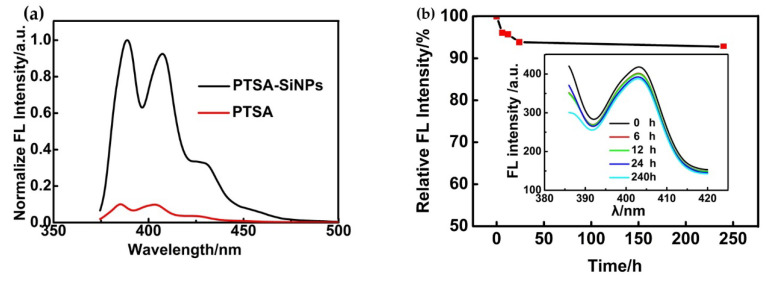
Fluorescence emission spectra of (**a**) PTSA-SiNPs and PTSA in H_2_O (concentration 10^−5^ M). Excitation of the two samples is at 376 nm. (**b**) The relative fluorescent intensity of the purified PTSA-SiNPs in H_2_O with the change in time (the insert image shows the fluorescence emission spectra of the purified PTSA-SiNPs in H_2_O at different times).

Fluorescence emission spectra of PTSA-SiNPs and PTSA in aqueous solution are shown in Figure 5a. The fluorescence emission spectrum of PTSA-SiNP dispersion shows two distinct peaks at 388 nm and 407.5 nm under 376 nm exciting light. Fluorescence intensity of the PTSA-SiNP dispersion is evidently higher than that of the PTSA solution. Compared with the original PTSA solution, the emission peaks of PTSA-SiNP dispersion have a slight red shift (about 4 nm). This is mainly due to the fact that the distance between the molecules decreases and the intermolecular force increases when the dye is doped into silicon nanoparticles, resulting in the formation of a polymer in the solution, which leads to the red shift of the luminescence wavelength [35]. In order to further determine the doping quality of the prepared PTSA-SiNPs, fluorescence leakage experiments are carried out. It can be seen from Figure 5b that there is a small amount of dye leakage in the first 6 h after the purified PTSA-SiNP sample is immersed in water. After 24 h, the fluorescence intensity of the PTSA-SiNPs gradually becomes stable, and the particles generally do not leak. The fluorescence intensity of the nanoparticles remains at about 92%. This effectively proves that PTSA can be well doped into SiNPs with PDADMAC as an intermediate because of the strong electrostatic interaction. 

#### 3.1.5. Fluorescence Lifetime 

**Figure 6:**
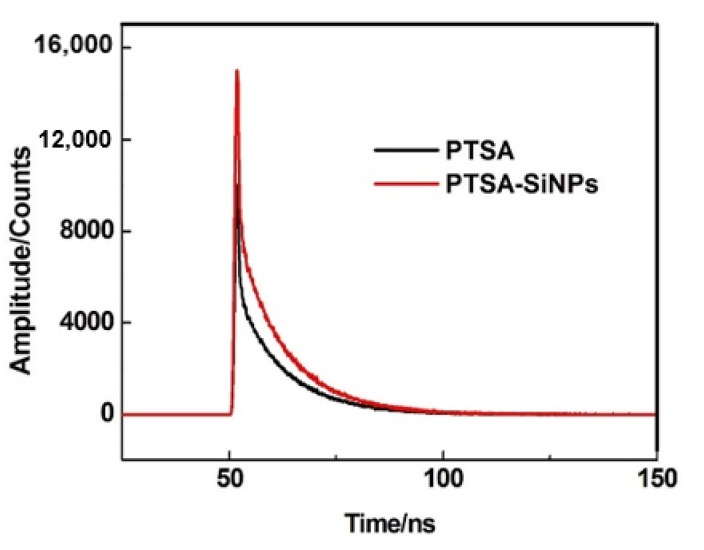
Fluorescence decay curves of PTSA-SiNPs and PTSA in aqueous solution (concentration 10^−6^ M).

The fluorescence lifetime is the time required for the fluorescence intensity to decay to 1/e of the initial intensity under the condition of determination. Fluorescence decay curves of PTSA-SiNPs and PTSA are shown in Figure 6. The curves of two substances are fitted to the mono-exponential fluorescence decay:

(1) $$I\left(t\right)=I_{0}\;\exp\left(-t/\tau \right)$$

The lifetimes of PTSA-SiNPs is 7.07 ns, which is slightly longer than that of PTSA (5.16 ns). That is to say, the doping of PTSA into SiNPs does not change the luminescence mechanism of the PTSA itself.

### 3.2. Application of PTSA-SiNPs in Anti-Counterfeit Ink

**Figure 7:**
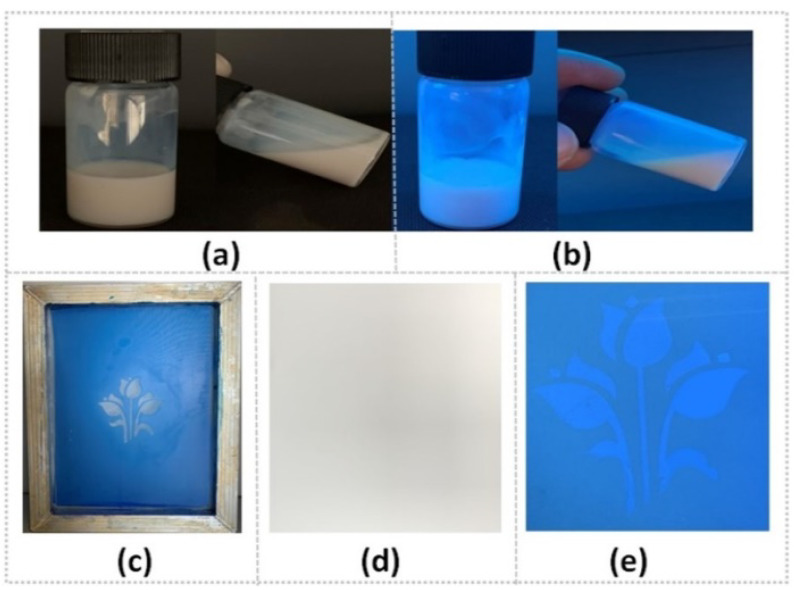
(**a**)The PTSA-SiNP ink under sunlight and (**b**) 365 nm UV light; (**c**) its screen-printing process of the printing plate and (**d**) printing pattern under sunlight and (**e**) 365 nm UV light.

Screen printing is one of the most widely used printing methods. The as-prepared PTSA-SiNPs are applied in the formulation of water-based fluorescent anti-counterfeit ink by screen printing. In Figure 7a,b, it can be seen that the appearance of the PTSA-SiNPs prepared ink is a milky white liquid under sunlight, and a blue fluorescence emulsion under UV light. The structure system of the PTSA-SiNP ink remains stable, without sedimentation, flocculation or stratification. The main reason is that the as-synthesized PTSA-SiNPs has good water compatibility and particle size uniformity, which is conducive to the stable existence of particles in the water emulsion dispersion system. The pattern of the ink printed on the paper is not visible under sunlight, but is clearly visible under UV light (Figure 7d,e). The ink can be well transferred by screen printing (Figure 7c) and can also realize a good fluorescence anti-counterfeit effect. 

In the system of water-based ink, the stability of pigments represents an important variable, crucial for their application potential [13]. Therefore, the as-prepared PTSA-SiNP material was applied to the water-based fluorescent anti-counterfeiting ink, and the fluorescence stability in the application process was tested.

#### 3.2.1. Heat Stability

**Figure 8:**
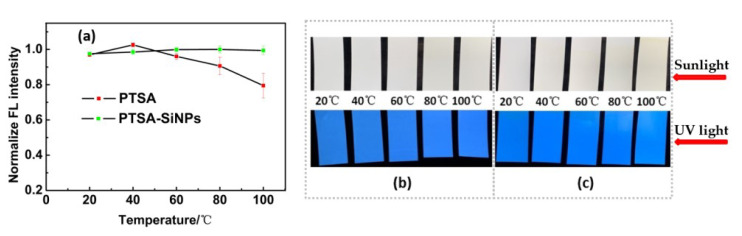
(**a**) Comparison of heat stability of the PTSA-SiNPs and PTSA anti-counterfeit ink; heat stability images of (**b**) PTSA-SiNPs and (**c**) PTSA anti-counterfeit ink samples under sunlight and UV light, respectively.

The drying of fluorescent water-based ink is realized by the evaporation of solvent, usually using hot air drying. In the drying process of water-based ink, fluorescent dye has to face at least two ordeals. One is that the fluorescence quenching may occur due to the narrowing of the distance between the dye molecules produced by solvent evaporation. On the other hand, with the increase in temperature, the collision frequency and deactivation probability of dye molecules increase. The heat resistance of fluorescent material is poor, resulting in poor fluorescence performance of the printing image. Heat resistance tests of the PTSA-SiNP ink are carried out in comparison with PTSA ink. The results can be seen in Figure 8. With the temperature increasing from 20 °C to 100 °C, the fluorescence intensity of the PTSA-SiNP ink samples remains stable, and its fluorescence brightness has no obvious change under UV light (Figure 8a,b). The fluorescence brightness of the PTSA ink samples does not change below 40 °C under UV light, but begins to decline when the temperature increases to 60 °C (Figure 8c). The maximum reduction of the fluorescence intensity is about 23% at 100 °C (Figure 8a). The drying temperature of the ink is usually controlled at about 60 °C. The higher the temperature, the higher the drying efficiency. PTSA-SiNP ink exhibits good fluorescent thermal stability. The main reason is that the matrix structure of SiNPs, as the physical crosslinking point in the system, limits the activity of dye molecules to a certain extent, inhibits the decomposition of dye molecules, and improves the heat resistance and fluorescent stability [36]. 

#### 3.2.2. Photostability 

**Figure 9:**
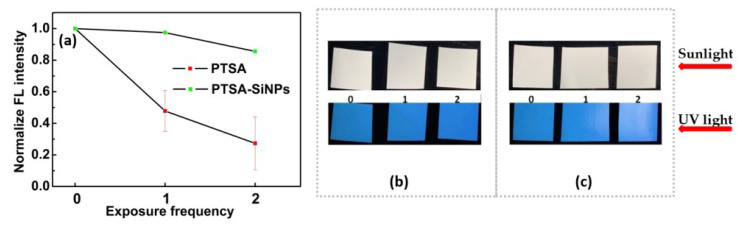
(**a**) Comparison of photostability of the PTSA-SiNP and PTSA anti-counterfeit ink; photostability photos of (**b**) PTSA-SiNP and (**c**) PTSA anti-counterfeit ink samples under sunlight and UV light (365 nm), respectively.

Photostability is also an important criterion to evaluate fluorescent anti-counterfeiting ink. The PTSA-SiNP ink samples are irradiated twice for 30 min each time while the PTSA ink samples are also tested. In Figure 9a, it can be seen that the fluorescence intensity of the PTSA-SiNP ink samples shows a reduction of about 13.5% after two times of irradiation, and that of the PTSA ink sample is reduced by about 73%. The PTSA-SiNP ink is much more photostable than the PTSA ink. The ink samples all exhibit natural color of printed paper under sunlight, as shown in Figure 9b,c. The PTSA-SiNP ink samples still present strong blue fluorescence under 365 nm UV light (Figure 9b), while PTSA ink samples present the photobleaching phenomenon. When PTSA is wrapped in silica matrix, the silicon shell acts as a protective layer to prevent the oxidation of dyes by UV, solvent and oxygen, thus preventing photobleaching and improving the photostability of dyes [37,38,39]. 

Due to the good photostability and thermal stability, the PTSA-SiNP water-based anti-counterfeit ink can not only be used to print paper-based commercial bills, packaging printed products and labels through screen printing, but it can also be expected to be used in commercial printing by heat-transfer printing and thermal ink-jet printing.

## 4. Conclusions

In summary, PTSA-SiNPs are successfully prepared by the reverse microemulsion method using cationic polymer PDADMAC as a bridge. The PTSA-SiNPs prepared by the reverse microemulsion method have the characteristics of a spherical shape, uniform particle size and good dispersibility, which is conducive to the stability of the ink dispersion system. By comparing the UV absorption, fluorescence emission intensity and fluorescence lifetime of the original PTSA, it is found that the fluorescence intensity of the PTSA loaded into SiNPs is significantly improved, and other luminescence properties are not significantly affected. The application of PTSA-SiNPs in the water-based anti-counterfeit ink shows that it has not only good fluorescent properties and an anti-counterfeit effect, but that it also exhibits excellent photostability and heat stability. All these results indicate that the as-prepared PTSA-SiNPs possess great potential as a pigment medium in water-based anti-counterfeit ink.

## Figures and Tables

**Table 1:** Formula of water-based anti-counterfeit ink components (wt.%).

Composition	PTSA-SiNP Ink	PTSA Ink
polyurethane waterborne emulsion	60	80
PTSA-SiNPs	15	−
PTSA	−	1
auxiliary	5	4
water	10–15	5–10
ethanol	5–10	5–10

## Supplementary Materials

The following are available online at https://www.mdpi.com/1996-1944/13/18/4074/s1, Figure S1: FT-IR spectra of (a) PTSA, (b) the product of PTSA-SiNPs prepared with PDADMAC, and (c) the product of PTSA-SiNPs prepared without PDADMAC.
