# Supplementary material for: Preparation of 1, 3, 6, 8-Pyrenesulfonic Acid Tetrasodium Salt Dye-Doped Silica Nanoparticles and Their Application in Water-Based Anti-Counterfeit Ink
Source: Materials (Basel). 2020 Sep 14;13(18):4074. doi: 10.3390/ma13184074 (PMC7560414; doi:10.3390/ma13184074)
Supplement: Supplementary file 1 [file materials-13-04074-s001.pdf]

materials

*Supplementary Materials*

# Preparation of 1, 3, 6, 8-Pyrenesulfonic Acid Tetrasodium Salt Doped Silica Nanoparticles and Its Application in Water-based Anti-counterfeit Ink

**Liyong Jiao [1,2,] *, Mengnan Zhang [2] and Houbin Li [1,] ***

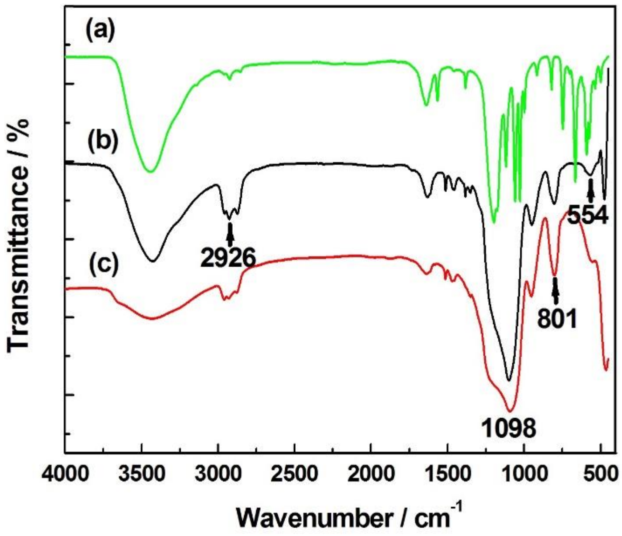


**Figure 1.** FT-IR spectra of (**a**) PTSA, (**b**) the product of PTSA-SiNPs prepared with PDADMAC, and (**c**) the product of PTSA-SiNPs prepared without PDADMAC.

*Materials* **2020**, *13*, x; doi: FOR PEER REVIEW 

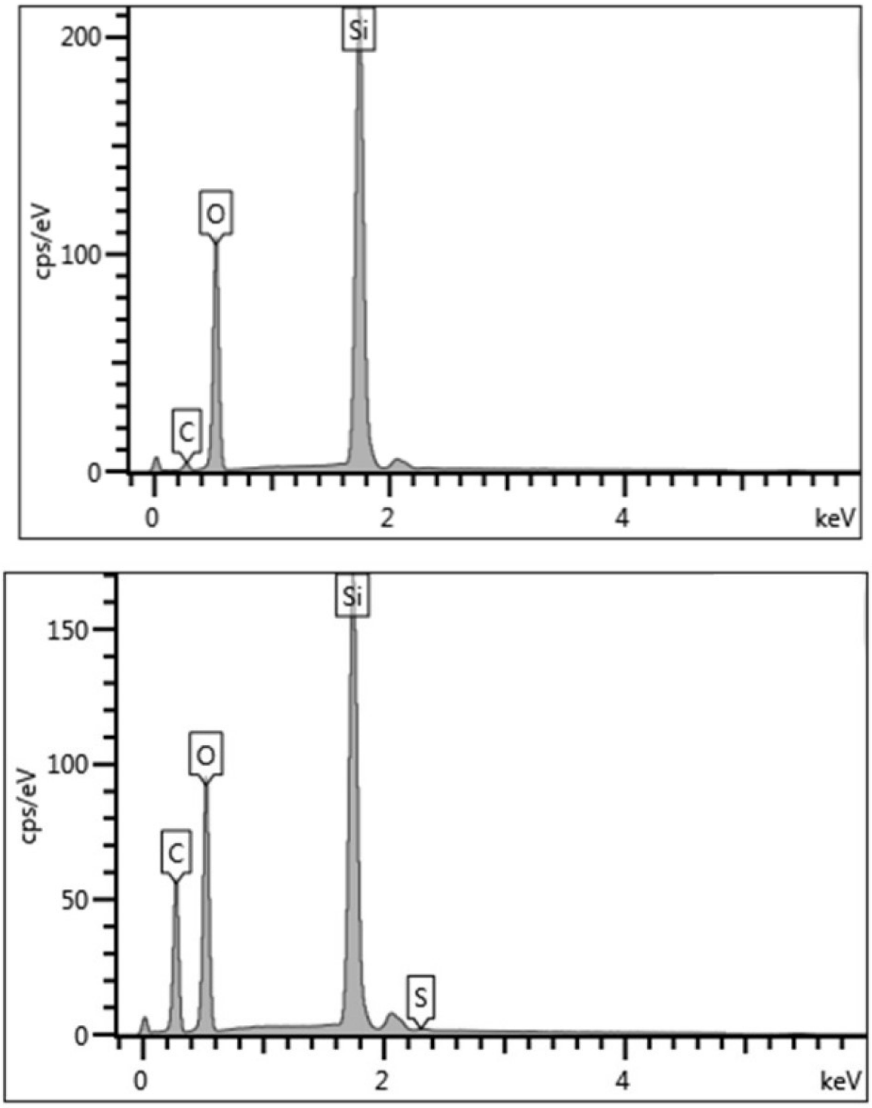


**Figure 2.** (**a**) EDS spectra of the product prepared by PTSA-SiNPs with PDADMAC (element mass distribution of PTSA-SiNPs is Si 13.32%, O 37.44%, C 49.54%, S 0.06%, respectively.); (**b**) EDS spectra of the product prepared by PTSA-SiNPs without PDADMAC (element mass distribution of PTSA-SiNPs is Si 34.60%, O 54.69%, C 10.72%, respectively.).
